# Retraction: Entropy-based financial asset pricing: Evidence from Pakistan

**DOI:** 10.1371/journal.pone.0347182

**Published:** 2026-04-14

**Authors:** The PLOS One Editors

The *PLOS One* Editors retract this article [1] due to concerns about:

- Non-compliance with PLOS policy on Authorship.
- Peer review reliability.
- The use of terminology that differs from standards in the field (e.g., “usual least squares” and “common least squares” instead of “ordinary least squares”, “straight relapse” instead of “linear regression”).

MM, RA, and YAK did not agree with the retraction. SW and SAK either did not respond directly or could not be reached.
